# Effect of Higher Education on Salaries

**Published:** 1919-01-11

**Authors:** 


### THE MATRONS' AND SISTERS' DEPARTMENT.

### EFFECT OF HIGHER EDUCATION ON SALARIES.

It has been shown that the introduction of a higher curriculum of training has the inevitable result of shortening the nurses' hours. Probationers cannot be kept on the stretch over ward work for an excessive number of hours if they are to acquit themselves with credit in the lecture and examination rooms. Sisters must in common decency have privileges corresponding to their superior status. And thus the average number of hours passed in ward duty comes to be lowered right through the establishment, with the result that the staff must be indefinitely increased. Beyond a certain fatigue point, intelligent labour begins to deteriorate. Higher education exposes this point to view.

The same process of levelling up may be noticed as the result of higher education in regard to salaries, but it begins at the other end. Reduction of hours affects probationers first and their superiors afterwards, for, indeed, it is usually probationers who are most glaringly overworked. Increase of salaries, effected through higher education, begins with the upper grades and may quite possibly not extend at all to the lower. Higher education in the training-school cannot possibly be brought about without the co-operation of a group of educated and really well-paid ward sisters, and heads of departments, working in concert with a matron of proved ability and a sister-tutor of high educational qualifications. The entire well-being of the training-school depends on the gathering together and maintenance of such a staff. Its monetary value to the profession cannot yet be properly estimated. The openings afforded through these posts to women of the highest possible attainments have hardly yet dawned upon nurses or upon the public. Salaries, even the best now offered, are far below what may be called the real market price of such women, far below what they are able to earn in other callings. And some faint-hearted members of the profession are found doubting whether hospitals supported by voluntary offerings or by municipalities can or ought to indulge in the "luxury" of an efficient and well-paid nursing staff.

Such doubters are perhaps oblivious of the extent to which the advance of science and the progress of social reform, involving, both of them, together with the future of the nation, depend on a thoroughly well trained nursing profession. The more keen the medical and surgical work of an institution, the higher value is set on the quality of the nursing. Physicians and surgeons alike are well aware that the lives of their patients and their own reputations lie to a large extent at the mercy of the nurses in the ward and in the theatre. Even more is this the case in private sick-rooms. Hence, we find the most eminent members of the medical profession foremost in their demand for better and more intelligent training among nurses. We find them eagerly contending for vast and costly improvements to the training-schools. We find them, not beating down salaries as they are falsely accused of doing, but exerting their influence, as at the Leeds General Infirmary the other day, in favour of alterations and improvements involving an expenditure of many extra thousands a year. We believe too that the outside public is gradually awaking to the perception that thoroughly good nurses can only be produced by training under educated women with high ideals and brilliant intuitions, and that such women are indispensable to the nation and ought to rank in the upper grades of the Civil Service.

